# The Red Squat Lobster *Pleuroncodes monodon* in the Humboldt Current System: From Their Ecology to Commercial Attributes as Marine Bioresource

**DOI:** 10.3390/ani13142279

**Published:** 2023-07-12

**Authors:** Ana Lucía Yapur-Pancorvo, Marco Quispe-Machaca, Fabián Guzmán-Rivás, Ángel Urzúa, Pepe Espinoza

**Affiliations:** 1 Facultad de Ciencias Veterinarias y Biológicas, Universidad Científica del Sur, Lima 150152, Peru; pespinoza@cientifica.edu.pe; 2 Environmental Resources Management (ERM), Lima 15046, Peru; 3 Programa de Doctorado en Ciencias Mención Biodiversidad y Biorecursos, Universidad Católica de la Santísima Concepción, Concepción 4090541, Chile; mquispe@doctorado.ucsc.cl (M.Q.-M.); fabian.guzman@ucsc.cl (F.G.-R.); 4 Centro de Investigación en Biodiversidad y Ambientes Sustentables (CIBAS), Universidad Católica de la Santísima Concepción, Concepción 4090541, Chile; 5 Departamento de Ecología, Facultad de Ciencias, Universidad Católica de la Santísima Concepción, Concepción 4090541, Chile; 6 Laboratorio de Ecología Trófica, Instituto del Mar del Perú, Esquina Gamarra y Gral. Valle s/n, Callao 070101, Peru

**Keywords:** crustaceans, marine food web, Peru-Chile current, marine ecology, fisheries, marine biotechnology, essential fatty acids, biomedical industry

## Abstract

**Simple Summary:**

The red squat lobster *Pleuroncodes monodon*, a key crustacean in the Humboldt Current System (HCS), plays an important role as predator and prey of several species of commercial interest. Off the Chilean coast, *P. monodon* presents benthic habits and constitutes the main resource of the industrial crustacean fishery. In contrast, off the coast of Peru, the red squat lobster exhibits pelagic habits, with knowledge gaps on traits of its ecology and no commercial fishery activities. Therefore, this review explores research conducted in the HCS, including ecological aspects and its use as a marine bioresource.

**Abstract:**

This study focused on gathering available information on *Pleuroncodes monodon*, a widely distributed crustacean in the Humboldt Current System. Off the Chilean coast, this species presents benthic habits and constitutes the main resource of the industrial crustacean fishery; many studies have been carried out on its life cycle during the last century. In contrast, off the coast of Peru, this species exhibits mainly pelagic habits, with latent information gaps on aspects of its life history and no commercial fishery activities, such as catching, taking or harvesting from the marine environment. *P. monodon* is an ecologically important species, as a source of energy for its predators, which include invertebrates, birds, marine mammals and fish of commercial interest. Thus, *P. monodon* seems to play a key role in this ecosystem, mainly as an intermediate link between top predators and the first links in the food chain. In addition, this species presents various adaptation strategies to the changing oceanographic parameters of the areas it inhabits, even tolerating hypoxic environments and great depths in order to avoid being predated. Likewise, from an economic viewpoint, it has a high commercial value as a marine bioresource with great potential in the pharmaceutical and food industries. Considering this, more studies must be carried out to corroborate the biological, ecological, and fishing importance of this species in order to generate efficient management measures and ensure a sustainable fishery.

## 1. Introduction

*Pleuroncodes monodon* [1], commonly known as the “red squat lobster” belongs to the Munididae family and can be found from 15° N in Mexico to 41° S in Chile [2]. Its wide distribution and great abundance throughout South America has led it to be considered as the main resource of the industrial fishery of demersal crustaceans in Chile since the 1950s, as well as a resource with high exploitation potential in Peru [3]. The vast majority of studies regarding aspects of its life cycle have focused on Chile, with few investigations carried out in Peru, mainly due to the fact that it is not currently considered a fishery resource there [4,5].

As an abundant species with a great diversity of habitats, it seems that *P. monodon* plays an important role as the predator and prey of several species, many of which are of commercial interest [5]. In this context, what do we know and what do we need to find out about the red squat lobster *Pleuroncodes monodon*? This article has focused on reviewing available information regarding *P. monodon* in the Humboldt Current System (HCS), including biological and ecological aspects, as well as its current and potential use as a marine bioresource.

For the search and collection of information, an exhaustive, structured and systematized review of indexed scientific articles was carried out on the Web of Science (WoS), Scopus and Google Scholar platforms. In addition, technical reports from governmental institutions were sought. The information and data gathered was subsequently analyzed and summarized for its inclusion in the present review.

## 2. Humboldt Current System

The HCS is one of the most productive areas in the world in terms of the biomass of small pelagic fish that inhabit it [6,7]. The HCS withstands strong pressures from fishing activities, such as the anchoveta *Engraulis ringens*, which represents 10% of the world catch, and the Chilean jack mackerel *Trachurus murphyi* off the Chilean coast, which represented 59% of the catches made in the Eastern South Pacific from 1983 to 2019 [8,9].

This System is characterized by the presence of the Humboldt Current, which originates from the Antartic Circumpolar Current off the Chilean coast (45° S) towards the north of Peru and Ecuador (4° S), as part of the South Pacific Gyre induced by the Coriolis effect [10]. It encompasses a wide range of diverse ocean habitats and subterranean currents of mainly cold-water masses that flow towards the equator and then the poles [11].

The HCS is also one of the four Eastern Boundary Upwelling Systems. It presents high spatio-temporal variability in various parameters and has areas of high nutrient concentration. The HCS also has a shallow oxygen minimum zone (OMZ) [6,7], which is the fourth most important worldwide, among the six most widely recognized [7,12].

The combined effect of the surface winds coming from the southeast and the Earth’s rotation generate the upwelling of cold deep waters rich in nutrients, such as nitrates (NO^3−^), phosphates (PO_4_^3−^) and silicates (SiO_2_^4+^), which sustain the first link in the food chain [13], with a productivity of <300 g C/m^2^/yr [14]. These upwelling areas tend to be more continuous off the Peruvian coast and northern Chile, while in southern Chile they tend to occur seasonally [14]. In particular, the most important nuclei off the coast of Peru are located at 4–5° S, 7.8° S, 11–12° S and 14–15° S [15], characterized by the constant coastal winds towards the equator, which persist throughout the year [16]. In addition, the main pelagic fisheries in Chile, such as the Chilean jack mackerel and anchoveta, are also close to the most important upwelling zones, around 20–22° S, 32–34° S and 36–38° S [14].

However, the HCS is also subject to climate variability at different time scales [13]. In particular, during El Niño Southern Oscillation (ENSO) events, large-scale changes are generated in the atmospheric and oceanic circulation, which lead to an increase in surface temperatures and a deeper oxycline, nutricline, and thermocline [17,18]. As a consequence, the decrease in the contribution of nutrients to the euphotic zone generates a reduction in primary production [16] that can affect the bioenergetic condition of individuals and their fundamental physiological processes, such as growth and reproduction [19].

On the other hand, the high oxygen consumption due to the decomposition of organic matter leads to the development of an oxygen minimum zone (OMZ) [12]. In the water column, oxygen deficiency also benefits biochemical denitrification processes and anaerobic oxidation of ammonia [20]. This OMZ presents a greater depth off the northern coast of Peru, being shallower in the central-south zone [21]. Off the Chilean coast, the OMZ decreases its extension and vertical intensity as it approaches the south, covering a depth range of 100 to 450 m [22]. Likewise, off the Peruvian coast, its depth reaches the continental shelf and the slope, causing the release of Fe (II) from the sediments into the water column, contributing to the productivity of the area [23].

The OMZ also seems to modulate the vertical structure of the pelagic habitat, leading several species of zooplankton and nekton (mainly crustaceans, dominated by *P. monodon* and followed by euphausiids and copepods) to carry out daily vertical migrations to anoxic depths to feed [24]. In fact, Antezana [25] affirmed the presence of a community structure based on a divided habitat in which species avoid coexisting spatiotemporally in the OMZ by migrating to and from this zone at different times.

**Figure 1:**
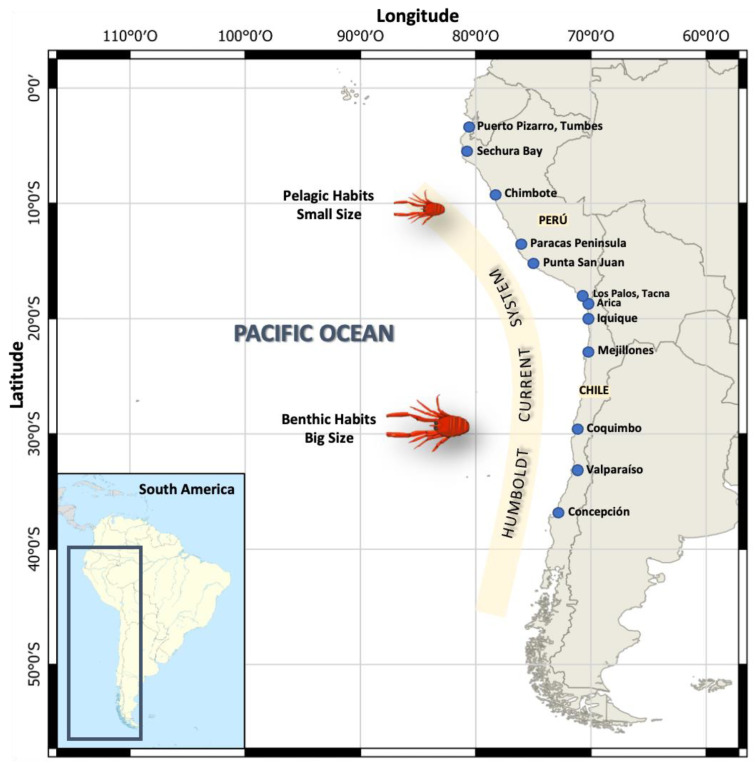
Geographic distribution of *Pleuroncodes monodon* off the Chilean and Peruvian coasts along the Humboldt Current System. Adults with two morphotypes of body size and contrasting lifestyle habits, as follows: Peru (pelagic habits and small size) and Chile (benthic habits and big size).

Considering that the coast of Peru goes from the parallels 0° to 18° S, and the coast of Chile extends from the parallels 17° to 56° S (Figure 1), it is also important to point out that the configuration of the continental shelf varies throughout these areas [26]. For example, off the Peruvian coast it tends to become narrower towards the south, while in the central zone, particularly in front of the district of Chimbote, department of Ancash (9°04′ S 78°35′ W), it becomes wider, reaching 70 nm and creating a favorable habitat for various pelagic resources [27]. On the other hand, off the coast of northern Chile, the continental shelf is narrow (~10 km) and becomes wider towards the south (~70 km) [28,29]. However, off the coast of Concepción (36–37° S) the Terrace of Itata can be found, which is the widest area of the continental shelf in central Chile, and where the highest values of primary productivity have been reported [26].

## 3. *Pleuroncodes monodon* throughout the HCS

Within the Decapoda order, the Munididae family is comprised of 356 species worldwide [30]. These crustaceans have some very particular morphological characteristics that allow them to live from surface areas to the abyssal bottom, while maintaining their abundance, such as anatomical adaptations of their carapace and chelipeds [31]. Within this family, *P. monodon* is distributed throughout the HCS off of Peru and Chile. Studies carried out on its distribution off the coasts of northern and central Peru (5–14° S) have classified its habits as coastal-pelagic [32], while in central and southern Chile it has been described as benthodemersal and benthic, according to the stage of its life cycle [33] (Figure 1).

An extensive analysis of its distribution that encompassed the entire Peruvian coastline (4–18° S), indicated that its distribution is directly influenced by the biomass of macrozooplankton, the depth of the oxycline and the distance to the edge of the platform, concluding that the most favorable habitat for *P. monodon* is represented by cold coastal waters [4]. Between 11° and 14° S off of central Peru, its presence has been reported at depths between 70 and 150 m, swimming in the water column or settled on the seabed, mainly during daylight hours and towards nightfall [34]. This species has also been reported at a depth of ca. 300 m on the continental margin off of Bahía de Concepción, in central Chile, by Gallardo et al. [35].

Considering the above, the real distribution of this species within the water column comes into question. As stated by Sanfuentes [5], *P. monodon* performs vertical migrations conditioned by the oxygen levels in the environment. In addition, Palash [36] indicated that it is usually located mostly in surface waters at night, while during the day it descends towards the OMZ. This vertical displacement represents a pattern of behavior of species that ascend towards the surface at nightfall to feed and descend at dawn to conserve energy and avoid predation [25,36].

Likewise, from the hatching period to recruitment, *P. monodon*, as any other planktonic larvae, is subject to changes in marine currents and advection [5,37]. Off Chilean coasts, it has been observed in a pelagic phase during its larval stage, adopting a benthic-demersal habit during its juvenile and adult stages [33]. North of 21° S and off the Peruvian coast, mainly pelagic habits have been reported, in response to the shallower depth of the oxycline, which is contrary to the situation south of 22° S, where benthic habits reaching 50–350 m of depth have been reported [33].

Similarly, *P. monodon* is usually distributed bathymetrically according to its reproductive phase and the conditions of its habitat [37,38]. In particular, off the Chilean coast, it has been reported inhabiting depths of 200–300 m in the autumn, and later migrating to depths of 70–200 m in the winter [39]. However, its distribution varies according to its life cycle and environmental parameters [40].

For example, from 22° S to 38° S, at a depth of 50 to 199 m, between the continental shelf and slope, an assemblage of species has been reported, including *P. monodon*, the yellow squat lobster *Cervimunida johni* and the bigeye flounder [40]. Similarly, Melo et al. [41] reported *P. monodon* together with the sea snail *Aenator fontainei*, as species associated with low oxygen contents and higher temperatures, under the influence of shallow Equatorial Subsurface Water (<300 m). The topographic variations of the environment also influence the distribution of *P. monodon* [26], as observed in the northern Chilean fishing unit between Coquimbo and Valparaíso with a high presence of males and females (ovigerous and non-ovigerous) at a depth of 150–170 m [42]. Considering this, these authors pointed out that the breadth of the continental shelf off of Concepción (36–37° S), together with the oceanographic characteristics of the area, could favor the retention of adult *P. monodon* individuals as aggregations.

During the first stages of their life cycle, they usually inhabit coastal waters with a greater abundance of phytoplankton, varying their distribution in the water column according to the availability of food, while during their most advanced stages they migrate to the bottom to establish their benthic juvenile phase [43,44]. Thus, benthic populations of *P. monodon* are usually associated with low temperatures, between 11 °C and 12 °C and low oxygen in the environment, while pelagic populations tend to be distributed above the oxycline with average temperatures between 15 °C and 16 °C [40]. In particular, off of Concepción (36–37° S), it has been shown that larvae tend to present a horizontal migration from the coast towards the continental slope as they develop from zoea to post-larvae [42] with zoea I and zoea II aggregations between 50 and 100 m [26], as was observed during the release of larvae in Coquimbo, when juvenile cohorts were found from 100 to 250 m deep [40].

Regarding the relationship that *P. monodon* has demonstrated with the oxygen concentrations in the environment, during its zoea stage, it has proven to be an oxyconformer, becoming an oxyregulator at the megalopa stage; however, at the juvenile and adult stages, oxyregulation is highly developed [26].

Off the Peruvian coast (∼3–18° S), *P. monodon* overlaps its habitat with the dark dolphin *Lagenorhynchus obscurus*, the long-beaked common *dolphin Delphinus capensis*, the bottlenose dolphin *Tursiops truncatus*, the anchoveta *Engraulis ringens*, the Chilean silverside *Odontesthes regia regia*, Patagonian squid *Doryteuthis gahi*, Peruvian sea catfish *Galeichthys peruvianus*, Chilean jack mackerel *Trachurus murphyi*, *Argonauta* spp., *Euphausia* sp., and Humboldt squid *Dosidicus gigas* [44,45,46]. However, the magnitude of the habitat overlap is unknown, which limits the analysis and the implications that this entails. What has been reported represents a challenge to be resolved in the future in order to understand the dynamics of interspecific relationships among these species.

## 4. Prey or Predator?

**Figure 2:**
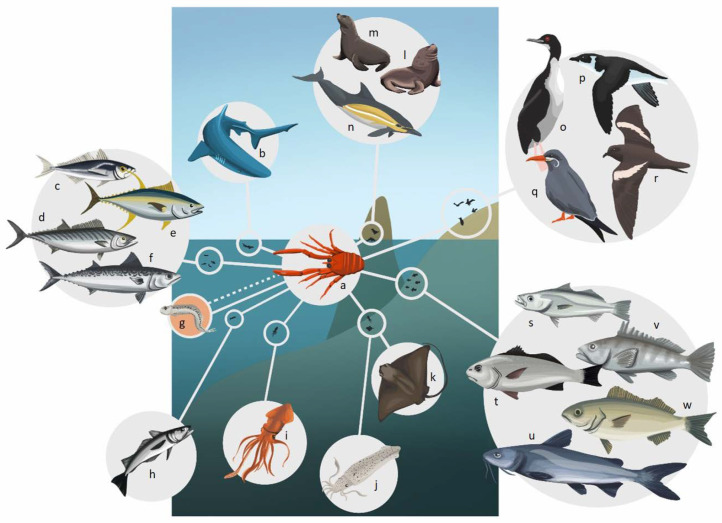
Predators of *P. monodon* off the Peruvian coast. (**a**) *P. monodon*, (**b**) *Prionace glauca*, (**c**) *Trachurus murphyi*, (**d**) *Sarda chilensis*, (**e**) *Thunus* sp., (**f**) *Scomber japonicus*, (**g**) *Engraulis ringens*, (**h**) *Merluccius gayi* peruanus, (**i**) *Dosidicus gigas*, (**j**) *Doryteuthis gahi*, (**k**) *Myliobatis chilensis*, (**l**) *Otaria byronia*, (**m**) *Arctocephalus australis*, (**n**) *Delphinus capensis*, (**o**) *Phalacrocorax bougainvillii*, (**p**) *Pelecanoides garnotii*, (**q**) *Larosterna inca*, (**r**) *Oceanodroma markhami*, (**s**) *Cynoscion analis*, (**t**) *Sciaena deliciosa*, (**u**) *Galeichthys peruvianus*, (**v**) *Paralabrax humeralis*, (**w**) *Isacia conceptionis*. The dotted line on (**g**) *Engraulis ringens* represents the possibility of mutual predation of eggs and larvae between this species and *P. monodon*, as has been reported by Gutiérrez et al. [32].

**Figure 3:**
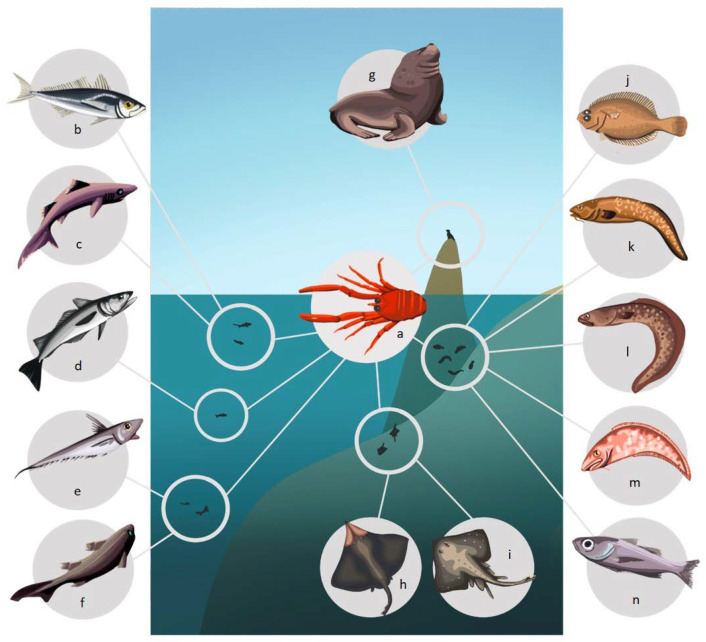
Predators of *P. monodon* off the Chilean coast. (**a**) *P. monodon*, (**b**) *Trachurus murphyi*, (**c**) Centroscyllium granulatum, (**d**) *Merluccius gayi gayi*, (**e**) *Coelorhynchus aconcagua*, (**f**) *Aculeola nigra*, (**g**) *Otaria byronia*, (**h**) *Zearaja chilensis*, (**i**) *Bathyraja* sp., (**j**) *Hippoglossina macrops*, (**k**) *Genypterus maculatus*, (**l**) *Genypterus blacodes*, (**m**) *Genypterus chilensis*, (**n**) *Epigonus crassicaudus*.

In this section, the trophic role of *P. monodon* in the HCS has been explored by reviewing its importance as prey, predator, and its interaction with potential competitors. Within this ecosystem, it is usually the prey of a large number of predators, among which fish, birds and mammals stand out, many of which are of commercial interest (Table 1) (Figure 2 and Figure 3).

Among its predators there are coastal, oceanic and benthic species. This coincides with the habits reported for this species on the HCS, where it usually presents benthic habits during most of its life cycle off of the coast of Chile [40], and mostly pelagic habits off of the coasts of Peru and northern Chile [32,76].

The first documented record of *P. monodon* as prey was made by Del Solar [52], who in a study carried out during 1930–1940 off the Peruvian coast, was able to detect its presence in the stomachs of various scombrids (such as the Eastern Pacific bonito *Sarda chiliensis*, tuna *Thunnus* spp. and the skipjack tuna *Katsuwomus pelamis*), which were in the area of influence of oceanic waters. Scombrids probably migrated to shore to feed or *P. monodon* migrated to ocean waters, which shows that *P. monodon* usually ventures into this area to feed, despite being a species that usually prefers coastal areas. As established by Del Solar [52], scombrids associated with oceanic waters can enter the coastal zone for short periods of time to have a greater source of food. Later, Konchina [77] defined this behavior as typical of facultative predators when studying the diet of the common hake *Merluccius gayi* and the jack mackerel *Trachurus symmetricus*, species that have different habitat preferences than the cold coastal waters typical of the HCS. However, recent studies have shown that the jack mackerel does not enter coastal waters due to the OMZ, which is expanding and limiting the volume of water for the entry of many other predators [78,79]; therefore, it is assumed that the Scombridae would not enter such an area, as previously established.

On the other hand, according to García-Godos et al. [73], ENSO events, by generating a shortage of anchoveta due to the incursion of warm waters in the coastal zone, favor a greater predation of *P. monodon* by other teleosts, as has been reported in the Peruvian rock seabass *Paralabrax humeralis* along the central coast of Peru [53]. Likewise, García-Godos et al. [66] reported that *P. monodon* was prey for the long-beaked common dolphin *Delphinus capensis*, along with other neritic and pelagic species, along the central and southern coasts of Peru (Table 1). Similarly, recent studies have shown that *P. monodon* is one of the most important prey for the Chilean jack mackerel *Trachurus murphyi* and chub mackerel *Scomber japonicus* along the entire Peruvian coastline (∼3–18° S) [53,63].

Off central Chile (25–36° S), it has been shown to be an important prey in the diet of the common hake *Merluccius gayi* [61,62].

*P. monodon* also constitutes an important prey for marine mammals and birds, as has been demonstrated in the Paracas Peninsula and in the Reserva Guanera Punta San Juan in Peru [71,73,80]. In northern Chile, in Arica (18°34′ S), Iquique (20°48′ S) and Mejillones (23°04′ S) *P. monodon* has been reported as the most important prey of the South American sea lion *Otaria byronia* [69] and as the dominant prey south of Iquique during the ENSO events of 1997–1998 and 2009–2010, which positively influenced its abundance [67].

Regarding the factors that influence its predation, a study developed by Kiko et al. [34], reported that *P. monodon* is vulnerable to predation due to its limited swimming capacity and reddish coloration, which stands out to its predators. Other factors that influence its predation include the presence of light equivalent to 1% that extends through the water column to a depth of 40 m and the oxycline manifested between 10 and 30 m of depth [75]. Therefore, it has been reported that to avoid predation *P. monodon* migrates towards the ocean bottom between hypoxic and anoxic environments during the day, reaching the continental slope, in further support that *P. monodon* is not strictly a coastal pelagic species [34].

Another possible strategy against predation is that *P. monodon* has been observed in video recordings crawling or partially buried in the substrate and inhabiting caves in large groups off the coast of central Chile (25–36° S) [81].

According to a report by Pauly and Christensen [82], *P. monodon* occupies a trophic position of 2.6, feeding mainly on primary producers, bacteria and debris, and classified as an opportunistic omnivore according to Lovrich and Thiel [83]. However, in a recent study its trophic position was identified as 3.6 and 3.8 according to its location off the southern and northern coasts of Peru, respectively [84]. Likewise, within their pelagic habitats, they have been identified as large filter feeders of large phytoplankton cells and small zooplankton [34], making this species a very important intermediate link between pelagic primary productivity and the lower layers of the water column [83]. Even though its benthic habitats are unknown, in Sechura Bay (5.6° S) it was observed feeding on debris through sediment resuspension [85]. Off the central zone of Chile, it was reported that it can prey on foraminifera, amphipods, zoeas, crustacean eggs, diatoms, organic matter and fish scales [86].

Likewise, it has been postulated that *P. monodon* can predate on the first stages of commercial species, among which the anchoveta is included, with which it shares a similar spatio-temporal distribution [4,87] (Figure 2). Along the Peruvian coast, both of these species most likely compete for food, including the possibility of mutual predation of eggs and larvae, which could potentially be negative [32,88]. However, compared to Peru, along the Chilean coast the anchoveta has a more extensive feeding area and is not as coastal as *P. monodon* [83].

Even though the predators of *P. monodon* include species that inhabit the water column in shallow and deep areas (Figure 2 and Figure 3), the magnitude of their predation and the impact they may have on its abundance is still unknown. Thus, taking into account that *P. monodon* represents an important link between lower and higher trophic levels in the trophic web, the potential effects that its increase or decrease in abundance could have on its predators or potential competitors must be considered.

In this regard, it has been postulated that *P. monodon* can predate on the first stages of commercial species, among which the anchoveta is included, with which it shares a similar spatio-temporal distribution [4,87]. Along the Peruvian coast, both of these species most likely compete for food, including the possibility of mutual predation of eggs and larvae (Figure 2), which could potentially be negative [32,88]. However, compared to Peru, along the Chilean coast the anchoveta has a more extensive feeding area and is not as coastal as *P. monodon* [84].

Currently, isotopic niche studies are currently underway (P. Espinoza; personal communication, July 2023); however, the available information on the trophic niche of *P. monodon* in the HCS are still scarce. Espinoza et al. [84] recommended analyzing the dietary habits of *P. monodon* in order to understand how the role of its pelagic or benthic phases varies, as well as that of other species with which it is involved. A recent study off the coast of Coquimbo and Concepción in Chile showed that juveniles are eminently filter feeders; this evidence was supported using trophic biomarkers from phytoplankton, small copepods and detritus [29]. However, further studies analyzing the dietary habits of *P. monodon* are still essential in order to understand current trophic relationships and thus implement fishery management plans with an ecosystem approach [89].

## 5. Reproduction and Bioenergetics

*P. monodon* is characterized by having a prolonged reproductive cycle with multiple annual offspring [90]. Off the coast of Concepción (Chile), it has been estimated that the reproductive potential of this species varies from 1808 to 33,966 eggs per laying [39,91], where individuals reach the adult stage after five larval stages [33].

Regarding the size of females that initially reach sexual maturity, Flores et al. [46] found LC50 values of ∼21.0 mm in the north and south of Chile, according to the functional criteria (histology), while according to physiological criteria, the LC50 values were 24.1 mm and 25.4 mm in the north and south, respectively. On the other hand, the same authors demonstrated a synchronous gonadal development among females of the population; however, the total number of offspring produced during the reproductive season is yet unknown.

Regarding the laying of eggs, a study carried out by Guzmán-Rivas et al. [92] in Chile proposed that this species could present an r-strategy (Please, refer to Jeschke et al. [93] and Reynolds [94] on r/k strategies and the concept of fast and slow life histories) during the summer and a k-strategy during the winter. Therefore, the authors pointed out that both the temperature and the availability of food in the environment influence their reproductive period. In Chile, the presence of ovigerous females was reported from February to December, with a greater abundance between May and October [39]. Similarly, between ∼36° S and ∼30° S peaks of ovigerous females in the benthic phase have been observed in the winter, coinciding with periods of high oxygen levels in the water column [40,95]. This characteristic coincides with the behavior of *P. monodon* populations in Costa Rica, where the reproductive period begins when the water temperature decreases and there is an upwelling event [91]. According to Pörtner and Gutt [96], this adaptation to the oceanographic conditions of the environment during the reproductive period allows *P. monodon* to maximize its fertility levels. However, a study carried out by Yannicelli and Castro [33] with ovigerous females collected on the south-central Chilean continental shelf showed that in the summer this species could experience better temperature conditions and food availability for hatching eggs, significantly improving development, growth and survival time. A study carried out along the Peruvian coast, from the coastal zone to approximately 100 nautical miles offshore, indicated that the highest percentages of females in a state of advanced maturity (stages 2 and 3) and with developed embryos (stage 4) was found during spring, indicating that the period of egg release or larval hatching takes place from this time of the year.

Considering the above, as a reproductive strategy, the larvae resort to the high capacity they have to tolerate certain periods of hypoxia, which allows them to disperse towards areas with more favorable oceanographic parameters until they reach the juvenile stage [33]. In Peru, the presence of *P. monodon* has been associated with intense upwelling events from Puerto Pizarro, Tumbes (03° S), to Los Palos, Tacna (18° S), favoring their reproductive period [97], as well as the availability of food by providing a constant source of nutrients that sustain primary productivity [98].

Tam et al. [99] indicated that the presence of a significant variation in the environmental conditions of the habitat could severely affect the reproductive period of *P. monodon*. For example, during the ENSO event of 1997–1998, there was a population decrease and a displacement towards the south, with its abundance later increased as a result of the expansion of the cold coastal waters [4]. On the other hand, events such as La Niña, are beneficial for the population of *P. monodon* because this species takes advantage of the cooling of the water masses to reproduce [4].

Likewise, oceanographic events indirectly affect the interannual and seasonal variability of the biochemical composition of *P. monodon* eggs. This has been evidenced by eggs presenting a higher concentration of lipids and a lower concentration of proteins during winter and vice versa during summer, resulting in a much higher energy content in the winter season [92]. This bioenergetic variability could also be influenced by the size and production of eggs, which can vary along a latitudinal gradient [100]. Similarly, it was reported that, in the winter, female bodies showed a higher percentage of lipids [101], saturated and polyunsaturated fatty acids [102], and their embryos presented increased concentrations of saturated and essential fatty acids (C18:2n6cis, C18:3n6 and C22:6n3) [101]. This is beneficial during hatching because it increases the probability of larvae survival in an environment with adverse variables [103]. These bioenergetic compounds, in the form of lipids and fats, favorably influence the nutrition, reproduction and growth of individuals [29,104].

It has been shown that the larvae that hatch from larger eggs in the winter are greater in size, have a higher dry weight and higher concentration of lipids and essential fatty acids [105]. This seems to be a reproductive adaptation strategy to ensure a high-energy reserve during seasons of lower temperature and less food availability [105]. Therefore, understanding the variations in the reproductive characteristics of this species and taking into account the fact that females must be managed in a particular way, it is important to establish better fishing models to guarantee the sustainable exploitation of *P. monodon* [100,106].

Regarding the mating behavior of this species, Espinoza-Fuenzalida et al. [95] showed that the mating behavior of *P. monodon*, which does not entail the need to molt, is beneficial, reducing energy costs during its reproductive period and the risk of being predated by its congeners or other species [107]. It is also advantageous for this species to produce more offspring in a shorter period of time [95]. Thus, identifying the reproductive biology of this species is essential to generate an efficient population management.

## 6. Genetic Aspects

The main morphological characteristics of *P. monodon* larvae were described by Fagetti and Campodonico [108] in order to distinguish them from their congener, *Pleuroncodes planipes*. However, Bianchi [109] stated that both species corresponded to *P. monodon*. Considering this, molecular analyzes (COI and 16S) were carried out, which confirmed that they belong to the same species [91].

Regarding its development, similar to other resident species, *P. monodon* consists of a population with a high gene flow throughout the HCS [110]. Kilada and Acuña [111] found differences in the growth parameters and sexual maturity of individuals collected off the northern and southern coasts of Chile. However, the authors attributed these spatial differences to the phenotypic response to habitat conditions during its benthic phase, with the oxycline representing the main oceanographic factor that could be exerting the most influence. To corroborate this, mitochondrial DNA studies indicated that the haplotypes of individuals with a pelagic life cycle and individuals with a benthic life cycle do not present genetic differentiation, sharing a common demographic history and a recent population expansion [110].

## 7. Fisheries

In the ‘90s this species became abundant off the coast of Peru, reaching values of up to 3.4 million tons [32]. Changes in ecosystem dynamics, such as the decrease of its predators may have generated the population increase of *P. monodon*, especially considering that the catches of sardines, one of its predators, decreased notably during that decade [112]. In Peru, *P. monodon* is not yet commercially exploited; however, the Ministry of Production (PRODUCE) has been promoting the extractive activity of this species since 2013 [4,113]. Even so, Instituto del Mar del Perú (IMARPE) [54] reported that from 1998 to 2007 this species was landed as bycatch for anchovy and other pelagic resources. Consequently, it is currently possible to investigate various parameters of its life cycle, taking into account that the population has not yet been exploited as a fishing resource as in [91].

Off the Chilean coast, *P. monodon* is a target species of trawling (23–39° S), comprising the Northern Fishery Unit (NFU) from the XV to the IV Region [3,7] and the Southern Fishery Unit (SFU) from the V to the VIII Region [29,114,115] as a fully exploited resource and under a recovering fishery regime, due to the high levels of overfishing that occurred between 1992 and 2000 [116,117]. The state of the resource is analyzed annually, and a potentially sustainable quota is established in relation to the stock assessments that have been carried out since 1993 [118]. Likewise, it has a closure per year (from January to March) and is subject to the measure of the maximum catch limit per shipowner [119], which has been established as a precautionary measure to prevent overfishing, and it has been effective in curbing overfishing. Between January and March, the fishery does not operate because the individuals are growing and molting. The percentage of landings of this species as a companion fauna of other target species is also regulated [120]. *P. monodon*, together with other demersal crustaceans, such as the Chilean nylon shrimp and yellow squat lobster, economically support artisanal and industrial fishing in Chile through the export of frozen and processed products [119]. However, it should be noted that *P. monodon* fishery activities lead to possible incidental catches of other species of economic importance, such as the Chilean common hake, the armed box crab, and the Patagonian grenadier *Macruronus magellanicus* [118].

However, in central Chile, it has been shown that when some predators, such as the Chilean common hake, Chilean jack mackerel and small pelagic fish are caught, a top-down effect is generated that is beneficial for *P. monodon* [121]. Considering this, the authors suggest considering trophic interactions when evaluating stocks and managing fisheries, bearing in mind that predation has a direct effect on the population dynamics of other commercially important resources.

Hernáez and Wehrtmann [91] found differences in egg production between exploited and unexploited populations of *P. monodon*; various intrinsic and extrinsic factors most likely cause this variability. Considering that the Chilean population is in the exploitation phase, while the Peruvian population is not, comparative studies could be carried out in order to clarify whether there are significant differences in the egg production of this species throughout the HCS.

## 8. Implications as a Marine Bioresource

*P. monodon* has recently been called “future interesting food”, classifying it as a potentially edible species even better than other commercial species due to its concentrations of EPA, DHA, ARA and micronutrients such as Cu, Fe, Mn and Zn [122]. However, in the same mentioned study, 1.15 ± 0.59 µg/g wet weight of cadmium in the edible tissue was also recorded, which far exceeds the maximum permissible limit established at 0.5 µg/g wet weight for crustacean muscle, according to the Peruvian National Fisheries Health Agency [123]. Thus, the potential risk to human health should not be ignored, as well as the mechanisms of bioaccumulation and biomagnification, which should be resolved as soon as possible considering their carcinogenic nature and liver damage and kidney disease potential [124]. On the other hand, in Chile, where it is an exploited commercial resource, *P. monodon* is used as fresh-frozen tail and is exported mainly to the United States, Germany and Japan [125].

This species is not only important from the fishing point of view it can also be highly used as a raw material. From an industrial point of view, there are efforts aimed at extracting pigments (such as carotenoids and astaxanthin) and enzymes (such as digestive proteases) for the production and ripening of cheeses [126,127]. In fact, studies carried out in Peru have already proven that *P. monodon* offers a high potential for obtaining astaxanthin and fatty acids (Omega 3), it is thus recommended to be included in rainbow trout diets [128]. Consequently, it is an important species for food production; it can be included as a partial or total substitute for fishmeal, having proven to have greater digestibility and greater efficiency of feed conversion and growth in prawn farming [127,129].

Another usable compound of *P. monodon* in Chile is chitosan, a biopolymer derived from chitin obtained from its exoskeleton [130,131]. This compound has been shown to be effective as an antibacterial agent against problematic pathogens for aquaculture such as *Vibrio alginolyticus*, *Vibrio parahaemolyticus* and *Lactococcus garviae*, due to its cationic properties, causing a membrane alteration effect [132,133,134].

The application of chitosan has also been found to remove heavy metals such as chromium, copper, mercury and lead from water [135,136]. Similarly, it has been used in wastewater treatment, generating clarification by destabilizing the oil/water emulsion [137]. The success of chitosan derives from its physicochemical characteristics, which give it chemical stability, high reactivity and excellent chelating behavior [135]. Currently, there is no industrial chain in Peru, linked to the fishing sector for the extraction of chitosan, only there some laboratory studies related to the extraction of bioactive lipids from *P. monodon* [138,139], despite the fact that there are studies in other parts of the world that show its relevance as a marine bioresource [140].

In the biomedical industry, chitosan derived from *P. monodon* shows excellent results in the recovery of skin tissues damaged by burns, ulcers and injuries, due to its anti-hemorrhagic and antimicrobial properties [136]. It can also be used as a biopharmaceutical product for immunological, antitumor and hemostatic treatments, among others [141].

## 9. Conclusions and Future Perspectives

*P. monodon* presents various ecological clues (i.e., a high physiological flexibility: “oxygen and thermal tolerance”) and biochemical attributes (i.e., rich in essential fatty acids, bioactive compounds, chitosan, and carotenoids pigments) that allow could be an excellent candidate for the biotechnological industry. Particularly, this species has a high commercial value as a marine bioresource with great potential in the pharmaceutical and food industries.

In turn, *P. monodon* plays a key role within the HCS. It is highly tolerant to variations in the oceanographic parameters of its habitat, presenting various adaptation strategies that allow it to continue developing and reproducing. From an ecological point of view, this species seems to be channeling energy from the lower links of the trophic chain and fulfilling an important role as a common prey for organisms of various taxa.

Comparative studies between the Chilean and Peruvian populations of *P. monodon* could provide interesting insights about this species, taking into account that only one of them is under pressure from industrial fishery activities. Even though Chile has carried out a vast number of studies, there are still information gaps that must be resolved, including the displacement patterns and connectivity according to the life stage of the individuals, the total number of offspring produced during the reproductive period, and that subsequently recruit to adult populations.

Likewise, taking into account that in Peru there is a growing interest in establishing the extractive activity of *P. monodon* for its use as a marine bioresource, it is imminent that studies be carried out to clarify aspects of its life cycle and the inter- species relationships with other commercially important organisms. In this way, efficient population management could be ensured from an ecosystem approach.

## Figures and Tables

**Table 1:** List of predators of *P. monodon* throughout the Humboldt Current System.

Taxonomic Class	Scientific Name	Common Name	Location	References
Chondrichthyes	*Myliobatis chilensis*	Chilean eagle ray	Peru	[45]
	*Prionacea glauca*	Blue shark	Peru	[47]
	*Zearaja chilensis*	Yellownose skate	Chile	[48]
	*Bathyraja* sp.	Ray	Chile	[48]
	*Aculeola nigra*	Hooktooth dogfish	Chile	[48]
	*Centroscyllium granulatum*	Granular dogfish	Chile	[48]
	*Coelorhynchus aconcagua*	Aconcagua grenadier	Chile	[48]
Actinopterygii	*Cynoscion analis*	Peruvian weakfish	Peru	[49]
	*Engraulis ringens*	Anchoveta	Peru	[50]
	*Merluccis gayi peruanus*	Peruvian hake	Peru	[51]
	*Sarda chiliensis chiliensis*	Eastern Pacific bonito	Peru	[52]
	*Thunnus* sp.	Tuna	Peru	[52]
	*Scomber japonicus*	Chub mackerel	Peru	[53]
	*Paralabrax humeralis*	Peruvian rock seabass	Peru	[54,55]
	*Sciaena deliciosa*	Lorna drum	Peru	[55]
	*Isacia conceptionis*	Cabinza grunt	Peru	[55]
	*Galeichthys peruvianus*	Peruvian sea catfish	Peru	[56]
	*Hippoglossina macrops*	Bigeye flounder	Chile	[57,58]
	*Genypterus maculatus*	Black cusk-eel	Chile	[58]
	*Genypterus blacodes*	Pink cusk-eel	Chile	[59]
	*Genypterus chilensis*	Red cusk-eel	Chile	[60]
	*Merluccis gayi gayi*	Chilean common hake	Chile	[61,62]
	*Epigonus crassicaudus*	Cardinalfish	Chile	[48]
	*Trachurus murphyi*	Chilean jack mackerel	Chile and Peru	[48,53,63]
Cephalopoda	*Dosidicus gigas*	Humboldt squid	Peru	[64,65]
	*Doryteuthis gahi*	Patagonian squid	Peru	[65]
Mammalia	*Delphinus capensis*	Long-beaked common dolphin	Peru	[66]
	*Arctocephalus australis*	South American fur seal	Peru	[67]
	*Otaria byronia*	South American sea lion	Chile and Peru	[68,69,70,71]
Aves	*Larosterna inca*	Inca tern	Peru	[72]
	*Oceanodroma markhami*	Markham’s storm petrel	Peru	[73]
	*Phalacrocorax bougainvilli*	Guanay cormorant	Peru	[72,74]
	*Pelecanoides garnotii*	Peruvian diving petrel	Peru	[75]

## Data Availability

Not applicable.
